# Pulmonary Inflammatory Response in Lethal COVID-19 Reveals Potential Therapeutic Targets and Drugs in Phases III/IV Clinical Trials

**DOI:** 10.3389/fphar.2022.833174

**Published:** 2022-03-29

**Authors:** Andrés López-Cortés, Santiago Guerrero, Esteban Ortiz-Prado, Verónica Yumiceba, Antonella Vera-Guapi, Ángela León Cáceres, Katherine Simbaña-Rivera, Ana María Gómez-Jaramillo, Gabriela Echeverría-Garcés, Jennyfer M. García-Cárdenas, Patricia Guevara-Ramírez, Alejandro Cabrera-Andrade, Lourdes Puig San Andrés, Doménica Cevallos-Robalino, Jhommara Bautista, Isaac Armendáriz-Castillo, Andy Pérez-Villa, Andrea Abad-Sojos, María José Ramos-Medina, Ariana León-Sosa, Estefanía Abarca, Álvaro A. Pérez-Meza, Karol Nieto-Jaramillo, Andrea V. Jácome, Andrea Morillo, Fernanda Arias-Erazo, Luis Fuenmayor-González, Luis Abel Quiñones, Nikolaos C. Kyriakidis

**Affiliations:** ^1^ Programa de Investigación en Salud Global, Facultad de Ciencias de la Salud, Universidad Internacional SEK, Quito, Ecuador; ^2^ Latin American Network for the Implementation and Validation of Clinical Pharmacogenomics Guidelines (RELIVAF-CYTED), Madrid, Spain; ^3^ Escuela de Medicina, Facultad de Ciencias Médicas de la Salud y de la Vida, Universidad Internacional del Ecuador, Quito, Ecuador; ^4^ One Health Research Group, Faculty of Medicine, Universidad de Las Américas, Quito, Ecuador; ^5^ Institut für Humangenetik Lübeck, Universität zu Lübeck, Lübeck, Germany; ^6^ Integrated Research and Treatment Center, Center for Sepsis Control and Care (CSCC), Jena University Hospital, Jena, Germany; ^7^ Heidelberg Institute of Global Health, Faculty of Medicine, University of Heidelberg, Heidelberg, Germany; ^8^ Latin American Network for Cancer Research (LAN-CANCER), Lima, Peru; ^9^ Centro de Investigación para la Salud en América Latina (CISeAL), Pontificia Universidad Católica del Ecuador, Quito, Ecuador; ^10^ Grupo de Bio-Quimioinformática, Universidad de Las Américas, Quito, Ecuador; ^11^ BIOscience Research Group, Quito, Ecuador; ^12^ Facultade de Ciencias, Universidade da Coruña, A Coruña, Spain; ^13^ Instituto Nacional de Investigación en Salud Pública, Quito, Ecuador; ^14^ Biotechnology Engineering Career, Faculty of Life Sciences, Universidad Regional Amazónica Ikiam, Tena, Ecuador; ^15^ Faculty of Medicine, Universidad de Las Américas, Quito, Ecuador; ^16^ Laboratory of Chemical Carcinogenesis and Pharmacogenetics, Department of Basic-Clinical Oncology, Faculty of Medicine, University of Chile, Santiago, Chile

**Keywords:** pulmonary inflammatory response, clinical trials, drugs, lethal COVID-19, single nucleus RNA sequencing

## Abstract

**Background:** It is imperative to identify drugs that allow treating symptoms of severe COVID-19. Respiratory failure is the main cause of death in severe COVID-19 patients, and the host inflammatory response at the lungs remains poorly understood.

**Methods:** Therefore, we retrieved data from post-mortem lungs from COVID-19 patients and performed in-depth *in silico* analyses of single-nucleus RNA sequencing data, inflammatory protein interactome network, and shortest pathways to physiological phenotypes to reveal potential therapeutic targets and drugs in advanced-stage COVID-19 clinical trials.

**Results:** Herein, we analyzed transcriptomics data of 719 inflammatory response genes across 19 cell types (116,313 nuclei) from lung autopsies. The functional enrichment analysis of the 233 significantly expressed genes showed that the most relevant biological annotations were inflammatory response, innate immune response, cytokine production, interferon production, macrophage activation, blood coagulation, NLRP3 inflammasome complex, and the TLR, JAK-STAT, NF-*κ*B, TNF, oncostatin M signaling pathways. Subsequently, we identified 34 essential inflammatory proteins with both high-confidence protein interactions and shortest pathways to inflammation, cell death, glycolysis, and angiogenesis.

**Conclusion:** We propose three small molecules (baricitinib, eritoran, and montelukast) that can be considered for treating severe COVID-19 symptoms after being thoroughly evaluated in COVID-19 clinical trials.

## Introduction

The severe acute respiratory syndrome coronavirus 2 (SARS-CoV-2), etiological agent of the coronavirus disease 2019 (COVID-19), has led to more than 425 million cases and more than 5.9 million deaths globally (WHO, 2021). Since the World Health Organization (WHO) declared the outbreak of COVID-19 as a pandemic, the novel coronavirus has been acquiring several mutations that not only increase its transmissibility rate but also mediates evasion of the host immune response and vaccination surveillance. Positive selection maintains amino-acid variants that increase virus fitness, whereas negative selection generally removes changes that reduce virus fitness (Lo Presti et al., 2020). For instance, some of the most predominant variants are capable of escaping monoclonal antibodies, partially eluding the polyclonal immune responses induced by previous infection or even allowing re-infections. It should be noted that recent improvements in immune escape are linked to mutations that alter the N-terminal domain (NTD) rather than the receptor-binding domain (RBD) of the spike (S) protein, where early and functionally important alterations predominated (Burioni and Topol, 2021). However, improved transmissibility, rather than immunoreaction or increased lethality, are considered as the main route for the virus to become fitter and more viable (Boehm et al., 2021). The variants that are being carefully monitored include B.1.1.7 (Alpha), B.1.351 (Beta), P.1 (Gamma), B.1.617.2 (Delta), and B.1.1.529 (Omicron) as variants of concern (VOCs); and C.37 (Lambda) and B.1.621 (Mu) as variants of interest (VOIs). It is expected that more variants will emerge over time that will need to be closely monitored, since they are a potential threat to public health (Burton and Topol, 2021; Harvey et al., 2021).

SARS-CoV-2 has the ability to infect human body cells through the angiotensin-converting enzyme 2 (ACE2) protein receptor (Mccallum et al., 2021). Lung homeostasis maintains a fine balance between tolerance mechanisms against non-pathogenic agents, pro-inflammatory immune system activation to fight off respiratory tract infections, and anti-inflammatory and pro-fibrotic processes to minimize tissular lesion and promote repair functions. Heterogeneous lung cell types mediate these complex mechanisms. The pulmonary alveolar epithelium is mainly composed of alveolar type I (AT1) and type II (AT2) cells, which allow the gas-exchange function and synthesize pulmonary surfactant factors, respectively (Wang et al., 2018). Airway epithelial cells are responsible for early pathogen recognition and production of pro-inflammatory cytokines and type I interferon (Yoo et al., 2013). Pulmonary endothelial cells have pleiotropic functions that range from gas interchange to regulating vascular tone and facilitating immune cell recruitment and diapedesis upon receiving pro-inflammatory stimuli (Niethamer et al., 2020). Mast cells are innate immune cells involved in defense and surveillance (Espinosa and Valitutti, 2018). Macrophages are key sentinel cells that detect pathogen invasion or tissue damage and initiate acute inflammatory processes (Biswas and Mantovani, 2014). Conventional dendritic cells (cDCs) are the bridge between the innate and adaptive immune responses as they constantly sample antigens from the airways and/or the infected lung tissue. Thus, cDCs migrate to T-cell areas of secondary lymphoid organs and present antigens to T lymphocytes thereby activating them (Schraml and Reise Sousa, 2015). Monocytes, subsets of leukocytes mostly originated from myeloid progenitors in the bone marrow, are able to differentiate into macrophages or dendritic cells in peripheral tissues (Murray, 2018). Natural killer (NK) cells eliminate infected cells by releasing perforin and granzymes or by death receptor signaling (FasL/Fas interactions and the subsequent induction of apoptosis) (Maucourant et al., 2020; van Eeden et al., 2020). NK cells can also release IFNγ upon activation, thereby contributing to naive T helper cell activation and differentiation (Culley, 2009). The CD4^+^ T helper cell population induce long-term cellular and humoral antigen-specific immunity (Peng et al., 2020). The cytotoxic CD8^+^ T cells are activated by specific pathogen or tumor-derived antigen presented on class I major histocompatibility complex (MHC I) molecules (Zhang and Bevan, 2011). Treg cells prevent autoimmune responses by suppressing the activation of conventional T-cells (Gladstone et al., 2020; Savage et al., 2020). B cells have a key role in the humoral adaptive immune response and are responsible for the production of antigen-specific immunoglobulins (Shuwa et al., 2021). Plasma blast cells are terminally differentiated populations of effector B cells that produce antibodies during initial exposure to a pathogen and mediate the protective effects of vaccination (Nutt et al., 2015). Fibroblasts produce cytokines and chemokines (Smith et al., 1997). Smooth muscle cells provide the main support for the vessel wall structure and regulate vascular tone to maintain intravascular pressure and tissue perfusion (Wang et al., 2015). Lastly, neuronal cells release neurotransmitters and neuropeptides that allow fast communication with immune cells, maintaining homeostasis and fighting infections (Blake et al., 2019).

Single-cell biology techniques has been widely implemented to study the cellular underpinnings of COVID-19 (López-Cortés et al., 2021). Scientific evidence through single-cell RNA sequencing (scRNA-seq) analyses of bronchoalveolar lavage fluid and blood from severe COVID-19 patients has revealed the effects of SARS-CoV-2 infection on immune responses and cytokine dysregulation (Wilk et al., 2020; Xu et al., 2020). Additionally, several autopsy studies examining formalin-fixed paraffin-embedded (FFPE) tissue sections extended our knowledge on virus organotropism. However, these studies were limited in their discovery potential due to tissue type, prolonged post-mortem intervals–affecting RNA quality–, and low-plex assays (Ackermann et al., 2020b; De Michele et al., 2020; Puelles et al., 2020). Since the respiratory failure is the main cause of death in severe COVID-19 patients and the host inflammatory response at the lung tissue level remains poorly understood, Melms et al. (2021) have published the single-cell lung atlas of lethal COVID-19 cases, and motivated by this study, we performed in-depth *in silico* analyses of snRNA-seq data; inflammatory protein-protein interactome (iPPI) network; functional enrichment analysis; and the shortest pathways to physiological phenotypes (cell death, inflammation, glycolysis, and angiogenesis) to reveal potential therapeutic targets and drugs in advanced-stage COVID-19 clinical trials.

## Methods

### Demographic Information of Donor Samples

The retrieved data from Melms et al. (2021) consisted of a cohort of 19 (100%) COVID-19 patients (12 males and 7 females) who died at a median age of 72 years. Of them, 13 (68%) were Hispanic or Latino, 7 (37%) had body mass index higher than 30.0 (obese and severely obese), and all cases had lungs, heart, kidneys or liver failure at time of death. On the other hand, the control cohort comprised 7 (100%) individuals (4 males and 3 females) with a median age of 70 years. Of them, 5 (71%) were white individuals, and 5 (71%) had body mass index between 25 and 29.9 considered as overweight. All information of donor samples is fully detailed in the Supplementary Table S1.

### Gene/Protein Sets

We have retrieved a total of 719 inflammatory response genes/proteins from the David Bioinformatics Resource (https://david.ncifcrf.gov/) (Huang et al., 2009) using the (GO) term: 0006954 inflammatory response. We have also retrieved the 332 human proteins physically interacting SARS-CoV-2 proteins evidenced by Gordon *et al* (Gordon et al., 2020). Both sets will allow us to perform multi-omics analysis to identify potential therapeutic targets and drugs to treat severe COVID-19.

### Single-Nucleus RNA Sequencing Data

We performed in-depth *in silico* analyses comparing the transcriptomics data of 719 genes involved in the inflammatory response between 9608 alveolar type I cells, 11341 alveolar type II cells, 7332 airway epithelial cells, 1845 B cells, 7586 CD4^+^ T cells, 3561 CD8^+^ T cells, 2814 cycling NK/T cells, 1083 dendritic cells, 5386 endothelial cells, 21472 fibroblast cells, 25960 macrophages, 1438 mast cells, 3464 monocytes, 2141 NK cells, 2017 neuronal cells, 5391 plasma cells, 1437 smooth muscle cells, 649 Treg cells, and 1788 other epithelial cells. The snRNA-seq database was taken from the ‘COVID-19 Studies’ section of the Single Cell Portal (https://singlecell.broadinstitute.org/single_cell/covid19), and the transcriptomics data of 116,313 nuclei was taken from ‘Columbia University/NYP COVID-19 Lung Atlas’ study (https://singlecell.broadinstitute.org/single_cell/study/SCP1219/columbia-university-nyp-covid-19-lung-atlas?cluster=UMAP&spatialGroups=--&annotation=cell_type_intermediate--group--study&subsample=all#study-summary) (Melms et al., 2021).

The criteria of the analysis of the lung transcriptomics data was the following: “all cells” as subsampling threshold, “cell type intermediate” as selected annotation, and ‘uniform manifold approximation and projection (UMAP)’ as load cluster. We adjusted the mRNA expression taking into account Z-scores ≤ -2 as underexpressed and Z-scores ≥ 2 as overexpressed. Additionally, we designed dot plots to visualize the percentage of cells expressing a certain gene, box plots to compare the mean Z-score across cell types, and scatter plots of 2D UMAPs to visualize the mean log normalized expression of a cluster of significantly expressed genes per subpopulation cell, and biological annotations across cell types.

### Functional Enrichment Analysis

We performed the functional enrichment analysis to validate the correlation between significantly curated signatures of expressed genes and biological annotations related to COVID-19 (Reimand et al., 2016; Raudvere et al., 2019). The enrichment was calculated using g:GOSt version e101_eg48_p14_baf17f0 (https://biit.cs.ut.ee/gprofiler/gost) to obtain significant annotations (Benjamini-Hochberg FDR q-value < 0.001) related to GO: biological processes, Reactome signaling pathways, the Kyoto Encyclopedia of Genes and Genomes (KEGG) signaling pathways, and Wikipathways (Ogata et al., 1999; Reimand et al., 2016; Slenter et al., 2018; Raudvere et al., 2019; Jassal et al., 2020). The functional enrichment is evaluated using the well-proven cumulative hypergeometric test whose main source of information is the Ensembl database (Cunningham et al., 2018). Finally, the expression of genes involved in significant annotations was visualized in scatter plots of lung cells, and the significant terms related to lethal COVID-19 were manually curated.

### Inflammatory Protein-Protein Interactome Network

The iPPI network with a highest confidence cutoff of 0.9 and zero node addition was designed between the human proteins involved in the pulmonary inflammatory response and the human proteins physically associated with SARS-CoV-2. To generate this network, we used the human proteome from the Cytoscape StringAPP (Doncheva et al., 2019), which imports protein interactions from the STRING database (Szklarczyk et al., 2015). The number of edges the node has in a network is represented by the degree centrality (López-Cortés et al., 2018; López-Cortés et al., 2020; López-Cortés et al., 2021), and it was calculated using the CytoNCA app (Tang et al., 2015). The network elements were organized through the organic layout producing a clear representation of complex networks, and the iPPI network was visualized through the Cytoscape software v.3.7.1 (Shannon et al., 2003). Finally, we ranked the inflammatory response proteins based on the highest confidence interactions with human-SARS-CoV-2 proteins. This network was validated by comparing their degree centralities with the inflammatory protein network by using the Mann-Whitney U test (*p* < 0.05).

### Shortest Pathways to Physiological Phenotypes Related to COVID-19

CancerGenNet (https://signor.uniroma2.it/CancerGeneNet/) is a bioinformatic tool curated by SIGNOR (Perfetto et al., 2016) that based on experimental information, allows to infer likely pathways of causal interactions linking proteins to physiological phenotypes (Iannuccelli et al., 2020). The shortest distance scores or paths from proteins to cell death, inflammation, glycolysis, and angiogenesis were programmatically implemented using the shortest path function of *igraph* R package (Iannuccelli et al., 2020). We calculated the shortest distance scores of positive regulation from the inflammatory response proteins with the highest confidence interactions to the human-SARS-CoV-2 proteins to physiological phenotypes related to COVID-19. Lastly, once the essential inflammatory response proteins were identified, we performed a multiple comparison test by using the Bonferroni correction (*p* < 0.05, and a 95% confidence interval) to analyze the association of these therapeutic targets among the cell death, inflammation, glycolysis, and angiogenesis phenotypes.

### Drugs Involved in Advanced-Stage COVID-19 Clinical Trials

The Open Targets Platform version 21.06 (https://www.targetvalidation.org) is a robust data integrator for visualization of potential drug targets involved in a variety of diseases including COVID-19 (Carvalho-Silva et al., 2019). This platform has developed the COVID-19 Target Prioritization Tool (https://covid19.opentargets.org/) that integrates molecular data from the ChEMBL database (Gaulton et al., 2017) to provide an evidence-based framework to support decision-making on potential drug targets for COVID-19. Lastly, this platform details phases III/IV clinical trials associated with therapeutic targets, modality, mechanism of action, phase, type of drug, and target class (Carvalho-Silva et al., 2019).

### Statistical Analyses

The identification of inflammatory response genes with significant expression from 116,313 nuclei belonging to 19 different lung cell types were prioritized by using Z-scores and *p*-values. Therefore, genes with Z-score ≥ 2 and two-tailed *p* < 0.001 indicated significant over expression, and genes with Z-score ≤ -2 and two-tailed *p* < 0.001 indicated significant under expression. Additionally, box plots compared the mean Z-score across cell types, dot plots demonstrated the percentage of cells expressing a certain gene, and scatter plots of 2D UMAPs visualized the mean log normalized expression of significantly expressed genes per subpopulation of cells. The functional enrichment of the significantly curated signatures of expressed genes elucidated biological annotations related to lethal COVID-19. The enrichment analysis was performed by using the g:GOSt tool which determines the most significant GO: biological processes, Reactome signaling pathways, KEGG signaling pathways, and Wikipathways with Benjamini-Hochberg FDR *q* < 0.001. The iPPI network takes into account the highest confidence interactions (cutoff = 0.9). We validated the network of inflammatory proteins with high-confidence interactions with human proteins physically associated to SARS-CoV-2 proteins comparing the degree centrality of them with the inflammatory protein network by using the Mann-Whitney U test (*p* < 0.05). Lastly, we performed a multiple comparison test by using the Bonferroni correction (significant level of *p* < 0.05 and a 95% confidence interval) to analyze significant differences of the shortest distance scores among physiological phenotypes related to COVID-19: inflammation, cell death, glycolysis, and angiogenesis.

## Results

### Single-Nucleus RNA Sequencing Data

**FIGURE 1:**
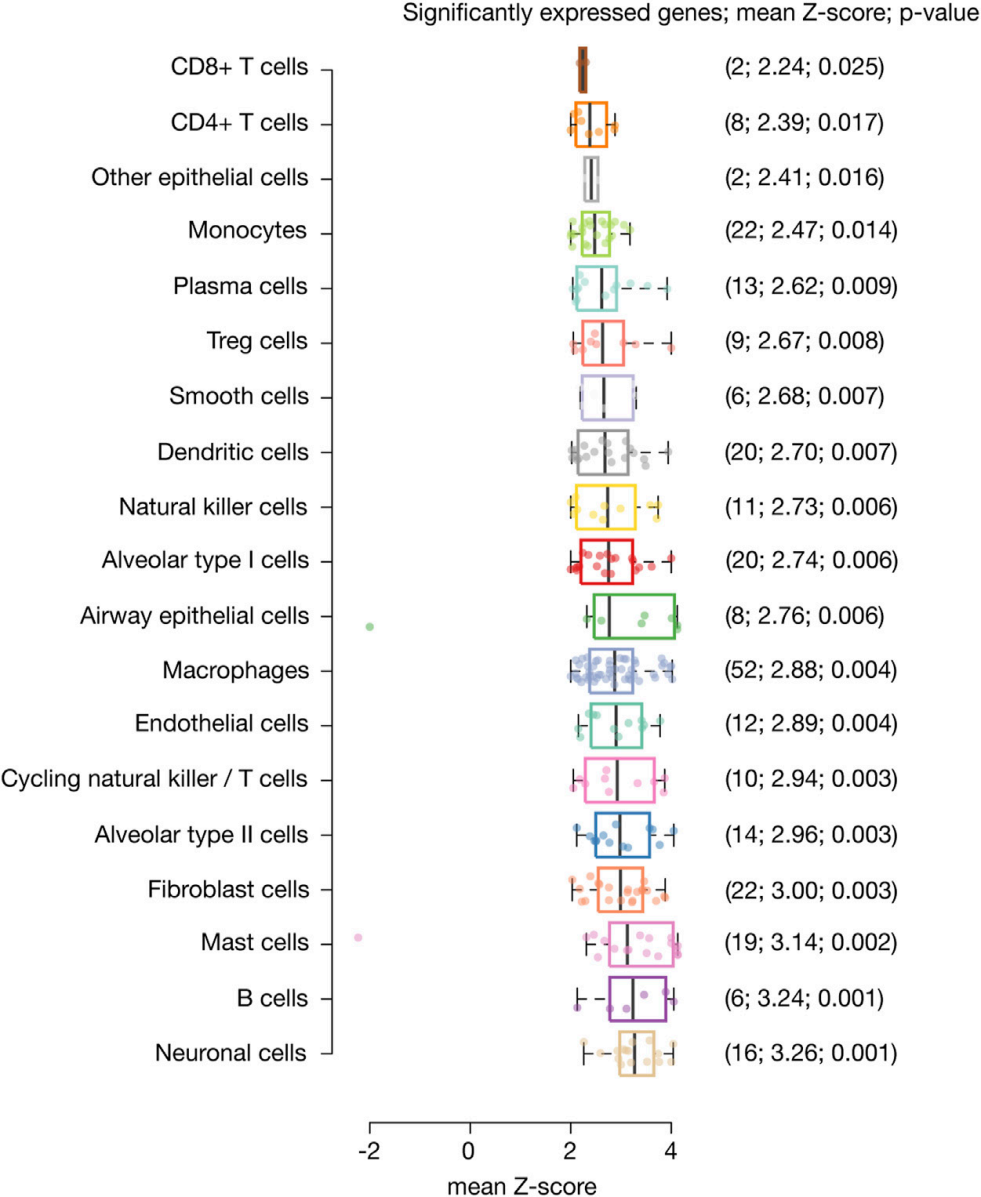
Significantly expressed genes across lung cell types. Box plots show lung cell types encompassed by significantly expressed genes, their mean Z-score and *p*-value. Neural cells were the cell type with the highest mean Z-score and most significant *p*-value, followed by B cells, mast cells, fibroblast cells, alveolar type II cells, cycling natural killer/T cells, endothelial cells, macrophages, airway epithelial cells, alveolar type I cells, natural killer cells, dendritic cells, smooth cells, Treg cells, plasma cells, monocytes, other epithelial cells, CD4^+^ T cells, and CD8^+^ T cells.

Single-nucleus biology is a powerful approach of omics medicine, needed to profile hard-to-dissociate tissues, that provides unprecented resolution to the cellular underpinnings of biological processes in order to find druggable targets for complex diseases (Slyper et al., 2020; Stephenson et al., 2021). Here, we identified 233 inflammatory response genes with significant expression in 116,313 nuclei belonging to 19 different lung cell types. Genes with the highest mean Z-score (3.26) and the most significant *p*-value (0.001) were identified in neural cells, followed by B cells (3.24; 0.001), mast cells (3.14; 0.002), fibroblast cells (3.0; 0.003), alveolar type II cells (2.96; 0.003), cycling NK/T cells (2.94; 0.003), endothelial cells (2.89; 0.004), macrophages (2.88; 0.004), airway epithelial cells (2.76; 0.006), alveolar type I cells (2.74; 0.006), NK cells (2.73; 0.006), dendritic cells (2.70; 0.007), smooth cells (2.68; 0.007), Treg cells (2.67; 0.008), plasma cells (2.62; 0.009), monocytes (2.47; 0.014), other epithelial cells (2.41; 0.016), CD4^+^ T cells (2.39; 0.017), and CD8^+^ T cells (2.24; 0.025) (Figure 1).

**FIGURE 2:**
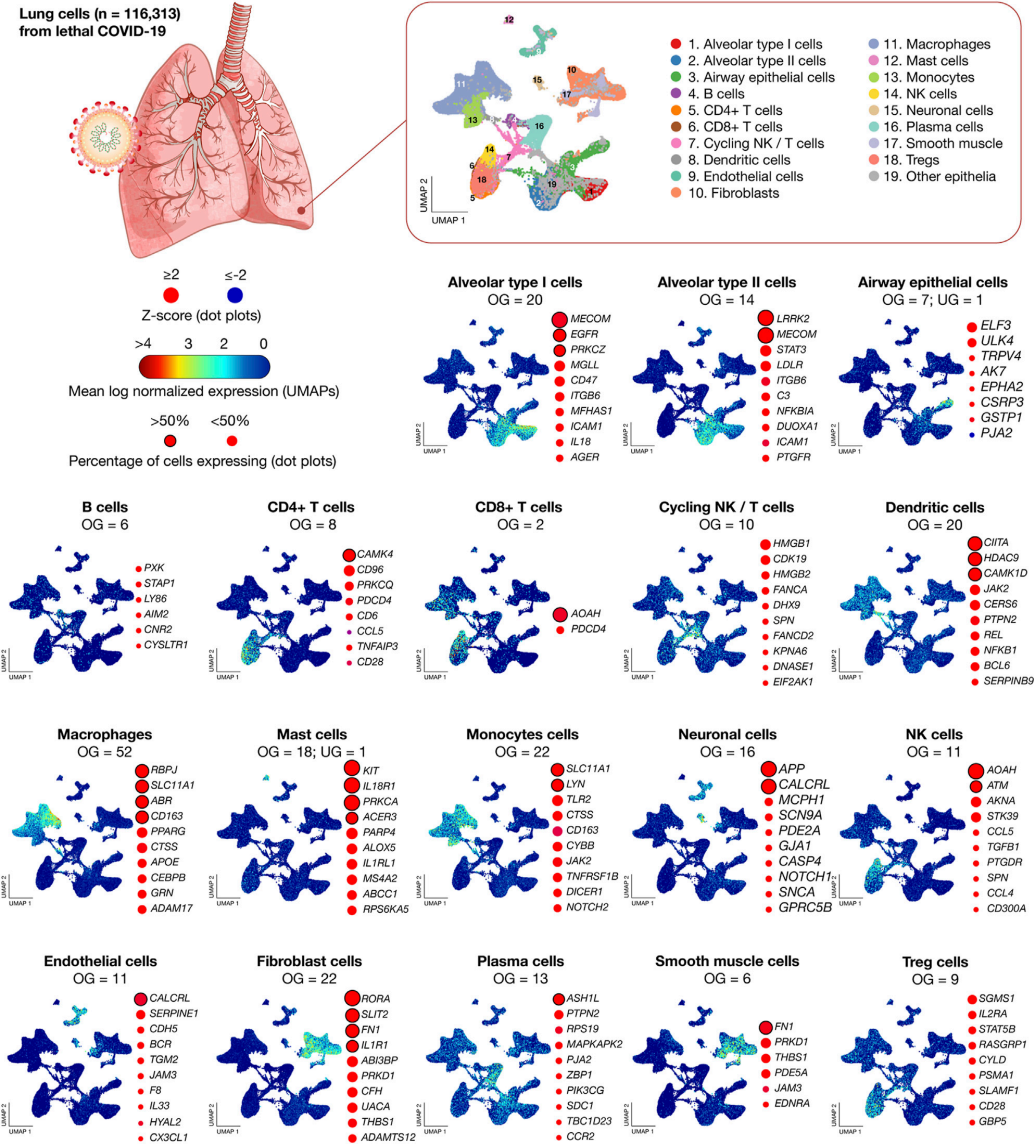
Transcriptomics data of 116,313 lung nuclei from 19 lethal COVID-19 patients. UMAPs show the mean log normalized expression of significantly expressed genes per lung cell type. Dot plots show the ranking of genes with the highest percentage of cells expressing. UMAP: uniform manifold approximation and projection for dimension reduction; NK, natural killer; OG, overexpressed genes; and UG, underexpressed genes.

Figure 2 shows scatter plots of significant mean log normalized gene expression and dot plots of genes with the highest percentage of cells expressing per lung cell type. *MECOM* has the highest percentage of cells expressing in alveolar type I cells, *LRRK2* in alveolar type II cells, *ELF3* in airway epithelial cells, *PXK* in B cells, *CAMK4* in CD4^+^ T cells, *AOAH* in CD8^+^ T cells, *HMGB1* in cycling NK/T cells, *CIITA* in dendritic cells, *RBPJ* in macrophages, *KIT* in mast cells, *SLC11A1* in monocytes cells, *APP* in neuronal cells, *AOAH* in NK cells, *CALCRL* in endothelial cells, *RORA* in fibroblasts, *ASH1L* in plasma cells, *FN1* in smooth muscle cells, and *SGMS1* in Treg cells. Lastly, the 26 inflammatory response genes significantly expressed in more that 50% of lung cells were *ABR*, *ACER3*, *AOAH*, *APP*, *ASH1L*, *ATM*, *CALCRL*, *CAMK1D*, *CAMK4*, *CD163*, *CIITA*, *EGFR*, *FN1*, *HDAC9*, *IL18R1*, *IL1R1*, *KIT*, *LRRK2*, *LYN*, *MECOM*, *PRKCA*, *PRKCZ*, *RBPJ*, *RORA*, *SLC11A1*, and *SLIT2* (Supplementary Table S2).

### Functional Enrichment Analysis

**FIGURE 3:**
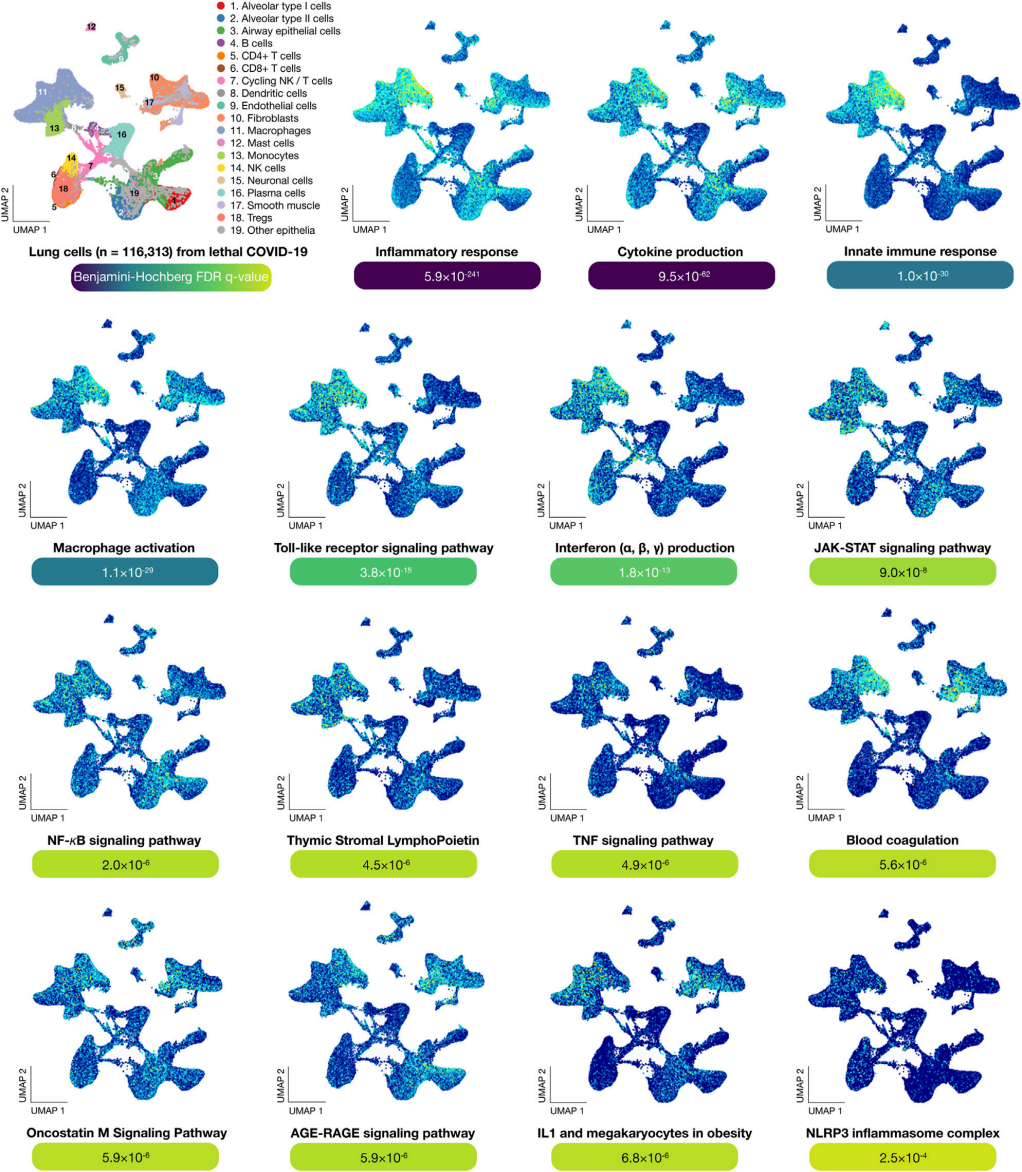
Functional enrichment analysis. UMAPs show the most significant genes per lung cell type involved in biological processes and signaling pathways. The most significant (Benjamini-Hochberg FDR q-value < 0.001) biological term was inflammatory response, followed by cytokine production, innate immune response, macrophage activation, Toll-like receptor signaling pathway, interferon production, JAK-STAT signaling pathway, NF-κB signaling pathway, thymic stromal lymphopoietin, TNF signaling pathway, blood coagulation, oncostatin M signaling pathway, AGE-RAGE signaling pathway, IL-1 and megakaryocytes in obesity, and NLRP3 inflammasome complex. UMAP: uniform manifold approximation and projection for dimension reduction.

This enrichment was performed using g:GOSt to obtain significant biological processes and signaling pathways related to lethal COVID-19 (Benjamini-Hochberg FDR *q* < 0.001) (Reimand et al., 2016; Raudvere et al., 2019). Figure 3 shows scatter plots of significantly expressed genes (*n* = 233) in lung cells of lethal COVID-19 autopsies. After a manual curation of biological annotations, the most significant GO terms were inflammatory response (5.9 × 10^−241^), cytokine production (9.5 × 10^−62^), innate immune response (1.0 × 10^−30^), macrophage activation (1.1 × 10^−29^), toll-like receptor signaling pathway (3.8 × 10^−15^), type I and II interferon production (1.8 × 10^−13^), the Janus Kinase (JAK)/Signal Transducers and Activators of Transcription (STAT) signaling pathway (9.0 × 10^−8^), NF-*κ*B signaling pathway (2.0 × 10^−6^), thymic stromal lymphopoietin (TSLP) (4.5 × 10^−6^), TNF signaling pathway (4.9 × 10^−6^), blood coagulation (5.6 × 10^−6^), oncostatin M signaling pathway (5.9 × 10^−6^), AGE-RAGE signaling pathway (5.9 × 10^−6^), IL-1 and megakaryocytes in obesity (6.8 × 10^−6^), and NLRP3 inflammasome complex (2.5 × 10^−4^) (Supplementary Table S3).

### Inflammatory Protein-Protein Interactome Network

**FIGURE 4:**
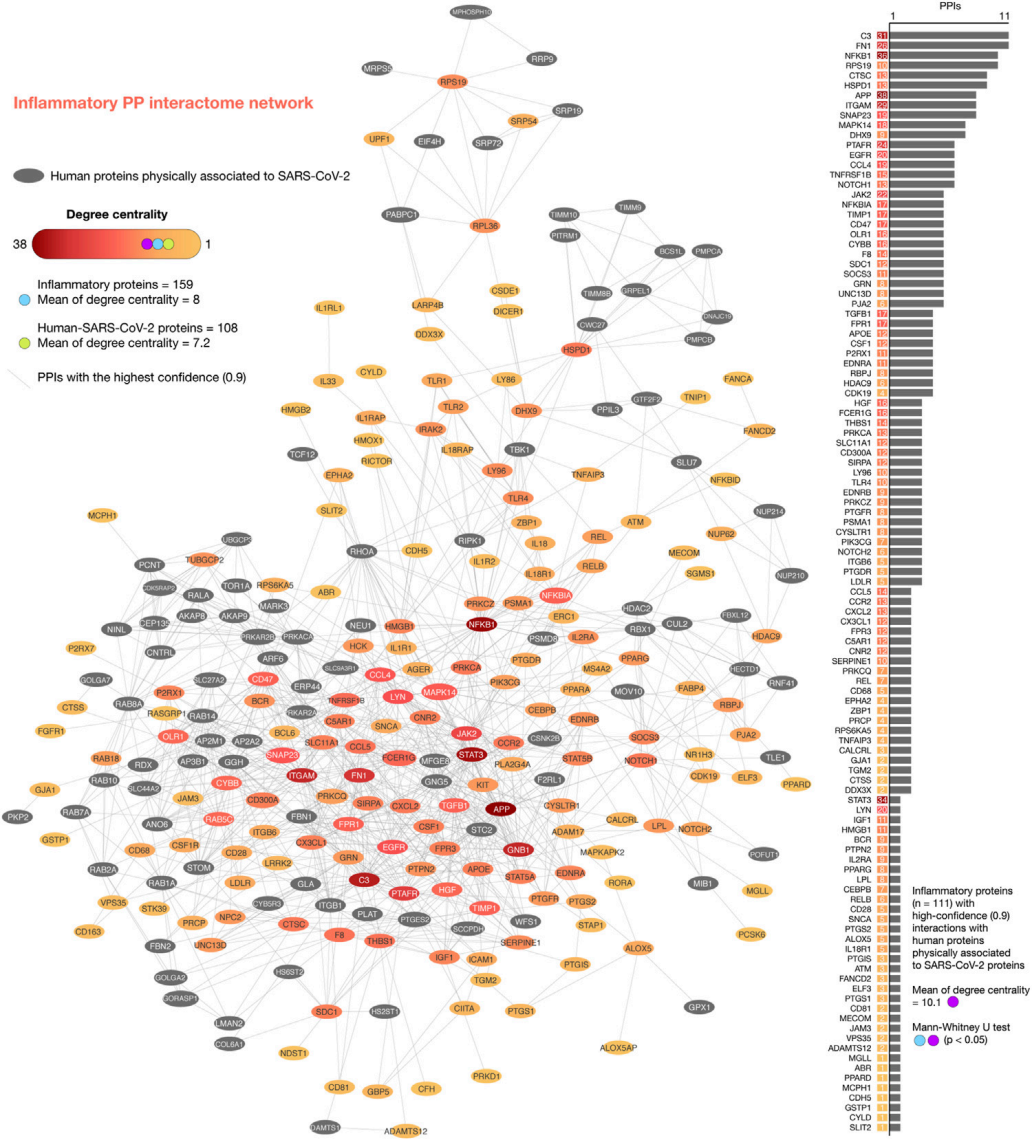
Inflammatory protein-protein interactome network. iPPI network was made up of 265 nodes and 2052 edges. Of them, 159 inflammatory response proteins had a mean of degree centrality of 8, and 108 human-SARS-CoV-2 proteins had a mean of degree centrality of 7.2. The top ten inflammatory response proteins with the highest degree centralities were APP, NFKB1, STAT3, C3, ITGAM, FN1, PTAFR, JAK2, EGFR, and LYN. The top ten human-SARS-CoV-2 proteins with the highest degree centralities were GNB1, GNG5, RHOA, ITGB1, STOM, RAB14, PRKAR2B, RAB8A, PRKACA, and ANO6. Additionally, the network of 111 inflammatory response proteins linked to human-SARS-CoV-2 proteins showed high-confidence interactions (cutoff = 0.9) and significantly higher degree centralities (Mann-Whitney U test, *p* < 0.05) in comparison to the complete iPPI network. The top ten proteins with the highest degree centralities were C3, FN1, NFKB1, RPS19, CTSC, HSPD1, APP, ITGAM, SNAP23, and MAPK14.

We generated the iPPI network encompassing 265 nodes and 2052 edges (Figure 4). Of them, 159 pulmonary inflammatory response proteins had a mean of degree centrality of 8 and 108 human-SARS-CoV-2 proteins had a mean of degree centrality of 7.2. The top ten inflammatory response proteins with the highest degree centrality were APP (38), NFKB1 (36), STAT3 (34), C3 (31), ITGAM (29), FN1 (26), PTAFR (24), JAK2 (22), EGFR (20), and LYN (20). The top ten human-SARS-CoV-2 proteins with the highest degree centrality were GNB1(29), GNG5 (25), RHOA (23), ITGB1 (22), STOM (20), RAB14 (20), PRKAR2B (17), RAB8A (17), PRKACA (17), and ANO6 (16). Additionally, 111 pulmonary inflammatory response proteins had the highest confidence interactions (cutoff = 0.9) with human-SARS-CoV-2 proteins, and a mean of degree centrality of 10.1, being the top ten: C3 (11 interactions), FN1 (11), NFKB1 (10), RPS19 (10), CTSC (9), HSPD1 (9), APP (8), ITGAM (8), SNAP23 (8), and MAPK14 (7) (Supplementary Table S4). Lastly, the network of inflammatory proteins linked to human-SARS-CoV-2 proteins showed significantly higher degree centralities in comparison to the inflammatory protein network (Mann-Whitney U test, *p* < 0.05).

### Shortest Pathways to Physiological Phenotypes Related to COVID-19

**FIGURE 5:**
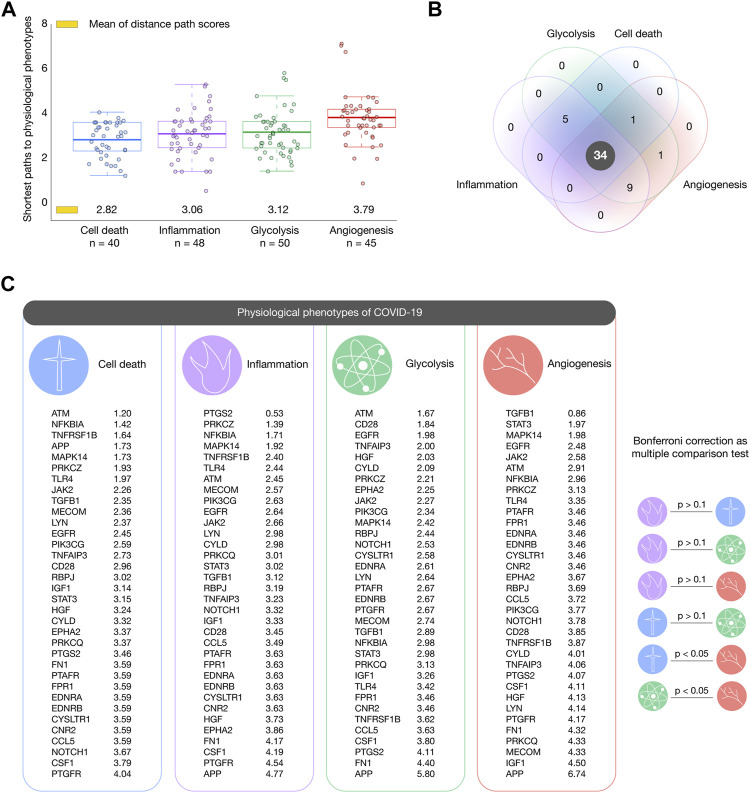
Shortest paths to cancer hallmark phenotypes. **(A)** Box plots encompassing inflammatory response proteins with the shortest mean of distance score per phenotype. Cell death was the phenotype with the shortest paths, followed by inflammation, glycolysis, and angiogenesis. **(B)** Venn diagram of inflammatory response proteins with shortest paths to hallmarks of cancer related to COVID-19. **(C)** Ranking of the most essential proteins with shortest paths to cell death, inflammation, glycolysis, and angiogenesis.

**FIGURE 6:**
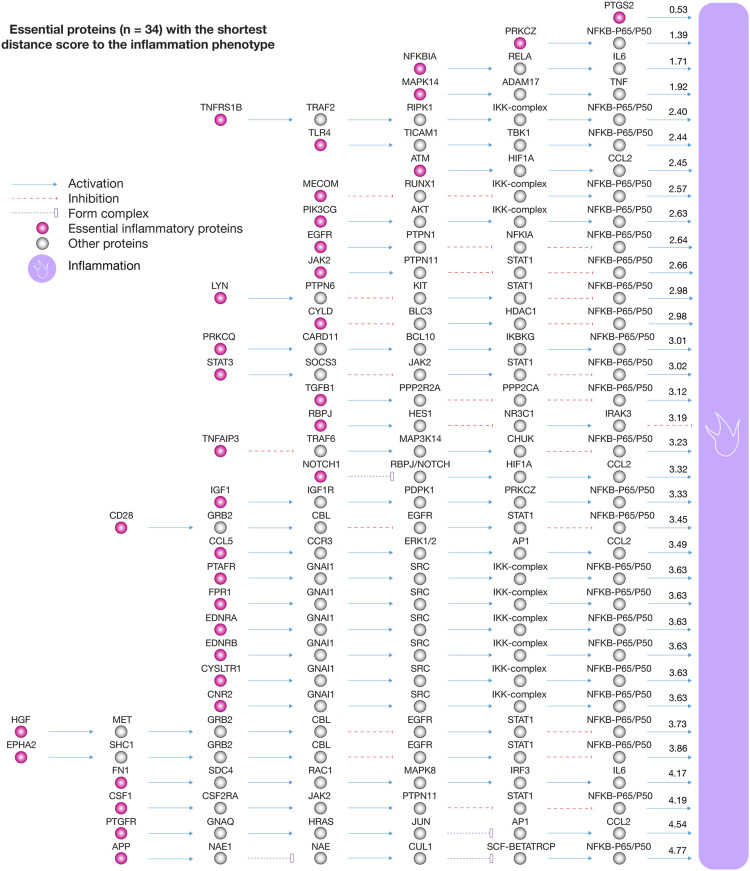
Essential proteins with the shortest distance score to the inflammation phenotype. The essential proteins with positive regulation to inflammation were PTGS2, PRKCZ, NFKBIA, MAPK14, TNFRSF1B, TLR4, ATM, MECOM, PIK3CG, EGFR, JAK2, LYN, CYLD, PRKCQ, STAT3, TGFB1, RBPJ, TNFAIP3, NOTCH1, IGF1, CD28, CCL5, PTAFR, FPR1, EDNRA, EDNRB, CYSLTR1, CNR2, HGF, EPHA2, FN1, CSF1, PTGFR, and APP.

We analyzed the 111 pulmonary inflammatory response proteins with the highest confidence interactions (cutoff = 0.9) to human-SARS-CoV-2 proteins in order to find the shortest pathways to inflammation, cell death, angiogenesis, and glycolysis (Iannuccelli et al., 2020). Figure 5A shows box plots encompassing proteins with the shortest distance scores to physiological phenotypes related to COVID-19. Cell death was the phenotype with the shortest mean of distance score (2.82), followed by inflammation (3.06), glycolysis (3.12), and angiogenesis (3.79). Figure 5B shows a Venn diagram integrating inflammatory proteins with shortest pathways to biological phenotypes related to COVID-19. We found 34 essential inflammatory response proteins with shortest pathways simultaneously to inflammation, glycolysis, cell death, and angiogenesis (Supplementary Table S5). Figure 5C shows the ranking of inflammatory response proteins with the shortest distance score to cell death, inflammation, glycolysis, and angiogenesis. Interestingly, the Bonferroni correction demonstrated that these 34 essential proteins do not have significant distance scores between physiological phenotypes (*p* > 0.1). The top ten essential proteins with shortest pathways of positive regulation to cell death were ATM (1.20), NFKBIA (1.42), TNFRSF1B (1.64), APP (1.73), MAPK14 (1.73), PRKCZ (1.93), TLR4 (1.97), JAK2 (2.26), TGFB1 (2.35), and MECOM (2.36). The top ten essential proteins with shortest pathways of positive regulation to inflammation were PTGS2 (0.53), PRKCZ (1.39), NFKB1A (1.71), MAPK14 (1.92), TNFRSF1B (2.40), TLR4 (2.44), ATM (2.45), MECOM (2.57), PIK3CG (2.63), and EGFR (2.64). The top ten essential proteins with shortest pathways of positive regulation to glycolysis were ATM (1.67), CD28 (1.84), EGFR (1.98), TNFAIP3 (2.00), HGF (2.03), CYLD (2.09), PRKCZ (2.21), EPHA2 (2.25), JAK2 (2.27), and PIK3CG (2.34). The top ten essential proteins with shortest pathways of positive regulation to angiogenesis were TGFB1 (0.86), STAT3 (1.97), MAPK14 (1.98), EGFR (2.48), JAK2 (2.58), ATM (2.91), NFKBIA (2.96), PRKCZ (3.13), TLR4 (3.35), and PTAFR (3.46) (Supplementary Table S6). Lastly, Figure 6 details all shortest pathways and distance scores of positive regulation from the 34 essential proteins to the inflammation phenotype.

### Drugs Involved in Advanced-Stage COVID-19 Clinical Trials

**FIGURE 7:**
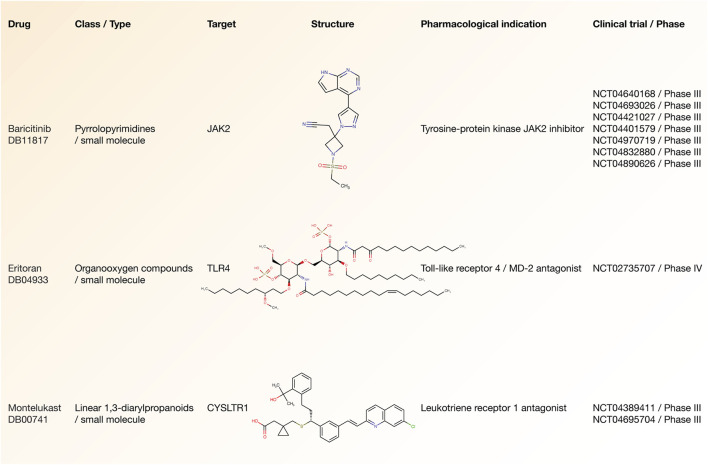
Drugs involved in advanced-stage COVID-19 clinical trials. Drug name, class type, druggable target, structure, pharmacological indication, and clinical trial number related to small molecules involved in phase III/IV clinical trials.

According to the Open Targets Platform, Figure 7 details the current status of COVID-19 clinical trials regarding to our essential inflammatory proteins (Carvalho-Silva et al., 2019). There are 3 drugs (small molecules) that are being analyzed in advanced-stage clinical trials (phases III and IV) and act on 3 essential inflammatory proteins. Baricitinib is a tyrosine-protein kinase JAK2 inhibitor that acts on the JAK2 protein and it is being studied in 7 phase III clinical trials (NCT04970719, NCT04401579, NCT04640168, NCT04693026, NCT04421027, NCT04832880, and NCT04890626). Eritoran is a Toll-like receptor 4/MD-2 antagonist that acts on the TLR4 protein and it is being studied in one phase IV clinical trial (NCT02735707). Lastly, montelukast is a cysteinyl leukotriene receptor 1 antagonist that acts on the CYSLTR1 protein and it is being studied in 2 phase IV clinical trials (NCT04389411 and NCT04695704) (Supplementary Table S7).

## Discussion

A wide spectrum of clinical features has been discovered in severe COVID-19. For instance, dyspnea, acute respiratory distress syndrome (ARDS) (Montenegro et al., 2020), respiratory failure, lung edema, severe hypoxemia, cardiac arrhythmias, lymphopenia (Terpos et al., 2020), hyperferritinemia, rhabdomyolysis, intravascular coagulopathy (Fogarty et al., 2020), and pulmonary thromboembolism (Rotzinger et al., 2020). Nowadays, it is known that SARS-CoV-2 not only causes respiratory tract infection, but also skin, kidneys, blood, and central neural system pathologies (Delorey et al., 2021). Therefore, it is imperative to continuously review the physiopathological mechanisms of the SARS-CoV-2 infection and the clinical manifestations, especially with the appearance of new genomic variants (Ortiz-Prado et al., 2020).

Single-cell biology provides a high quality resolution to the cellular underpinnings of biological processes in order to find therapeutically actionable targets (Gawel et al., 2019; Stephenson et al., 2021). Melms *et al* have previously published the single-cell lung atlas of lethal COVID-19 (Melms et al., 2021). Motivated by this study, we performed in-depth *in silico* analyses comparing the transcriptional data of 719 inflammatory response genes across 19 lung cell types belonging to COVID-19 autopsies. Regarding lung cell types with significantly expressed inflammatory genes, neuronal cells had the highest mean Z-score and significant *p*-value (3.26; 0.001), followed by B-cells (3.24; 0.001), mast cells (3.14; 0.002), fibroblast cells (3.00; 0.003), alveolar type II cells (2.96; 0.003), cycling natural killer/T cells (2.94; 0.003), endothelial cells (2.89; 0.004), macrophages (2.88; 0.004), airway epithelial cells (2.76; 0.006), alveolar type I cells (2.74; 0.006), natural killer cells (2.73; 0.006), dendritic cells (2.70; 0.007), smooth cells (2.68; 0.007), Treg cells (2.67; 0.008), plasma cells (2.62; 0.009), monocytes (2.47; 0.014), CD4^+^ T cells (2.39; 0.017), and CD8^+^ T cells (2.24; 0.025). Subsequently, the functional enrichment analysis of the 233 significantly expressed inflammatory genes showed that the most significant biological annotations were inflammatory response, cytokine production, innate immune response, macrophage activation, TLR signaling pathway, type I and II interferon production, JAK-STAT signaling pathway, NF-*κ*B signaling pathway, TNF signaling pathway, blood coagulation, oncostatin M signaling pathway, IL-1 and megakaryocytes in obesity, and the NLRP3 inflammasome complex (Table 1).

**TABLE 1:** Relevant response mechanisms found in significant biological annotations of COVID-19.

Biological Annotations
Type I and III IFNs
• Innate immunity is the first line of defense against SARS-CoV-2. The innate immune system is activated through TLR signaling. TLR3 is more abundant in NK cells, whereas TLR4 is more common in macrophages (Diamond and Kanneganti, 2022).
• Pattern recognition receptors (PRRs) activate transcription factors, such as NF-*κ*B, AP-1, and interferon regulatory factors (IRF3 and IRF7) that induce pro-inflammatory cytokines and type I interferon (Boechat et al., 2021).
• Type I IFNs are responsible for inducing the JAK-STAT signaling pathway to activate IFN-stimulated genes and promote the “anti-viral state” in the infected organism (Channappanavar et al., 2019).
Type I and III IFNs
• Cytokines involved in the immunological response against SARS-CoV-2 (Lopez et al., 2020).
• Zhang *et al* concluded that genetics may determine the clinical course of SARS-CoV-2 infection identifying mutations in genes involved in the regulation of type I and III IFN immunity (Zhang et al., 2020).
• Bastard *et al* identified high titers of neutralizing autoantibodies against type I IFN-α2 and IFN-ω in 10% of severe COVID-19 patients (Bastard et al., 2020).
• Inborn errors of immunity of type I IFN immunity, and pre-existing auto-antibodies neutralizing type I IFNs appear to be strong determinants of critical COVID-19 pneumonia in 15-20% of patients (Qian et al., 2022).
Macrophages
• Produce high amounts of pro-inflammatory cytokines in ARDS patients, those who then enter to massive pro-inflammatory state known as cytokine storm or macrophage activation syndrome (Otsuka and Seino, 2020).
• IL-6 plays a main role in COVID-19 severity, while TNF-α and IL-1β trigger the NF-*κ*B signaling pathway (Rowaiye et al., 2021).
• The overexpression of cytokines (i.e., TNF-α, IL-2, IL-10, IL-1, and IL-6) leads to development lung damage, cell death, severe pneumonia, ARDS, lung fibrosis, local or systemic thrombosis and multiple organ failure (Mustafa et al., 2020).
TNF
• The TNF-α-NF-*κ*B axis is considered as a potential therapeutic target for COVID-19 (Catanzaro et al., 2020).
• SARS-CoV-2-mediated NF-*κ*B activation has been observed in macrophages of liver, kidney, lung, central nervous system, cardiovascular system, and gastrointestinal system. This causes a chronic production of TNF-α, LT-α, LT-β, GM-CSF, IL-1, IL-2, IL-6, IL-12, and chemokines, leading to clinical manifestations (Hariharan et al., 2021).
• Catanzaro *et al* suggested that inhibiting the TNF-α-NF-*κ*B axis may prevent pulmonary complications in COVID-19 patients (Catanzaro et al., 2020).
JAK-STAT signaling pathway
• The cytokine signaling depends on the JAK and STAT transcription factors which are phosphorylated and activated upon cytokines binding to their receptors (Luo et al., 2020).
• Inhibition of the JAK-STAT signaling pathway seems as promising approach to prevent cytokine storm in severe cases or in patients with comorbidities that express high levels of inflammatory markers such as of IL-6, TNFα, IL-17a, GM-CSF, and G-CSF (Rojas and Sarmiento, 2021).
• The JAK/STAT signaling pathway is also an important mediator of the immune response that leads to viral infection clearance and prolonged inhibition of the pathway could lead to immunosuppression and persistent infections (Satarker et al., 2020).
Blood coagulation
• Tang *et al* reported that 71.4% of non-surviving COVID-19 patients met the criteria for disseminated intravascular coagulation and presented high levels of coagulation-related biomarkers such as D-dimer and fibrin degradation products (Tang et al., 2020).
• Exacerbation of inflammatory cytokine secretion promoting proliferation of megakaryocytes, lymphocyte cell-death, hypoxia, endothelial damage and the association between neutrophil extracellular traps and autoantibodies seem to be involved in the abnormal thrombotic events observed in COVID-19 (Biswas et al., 2021; Blasco et al., 2021).
Oncostatin M signaling pathway
• Oncostatin M stimulates CCL1, CCL7 and CCL8 in primary human dermal fibroblasts at a faster kinetics than IL-1β or TNF-α (Hintzen et al., 2008).
• Oncostatin M was proposed as a new mortality biomarker in patients with acute respiratory failure supported by venous-venous extracorporeal membrane oxygenation (Setiadi et al., 2020).
• Oncostatin M induces obesity and insulin resistance conditions in COVID-19 patients (Sanchez-Infantes and Stephens, 2021).
Obesity
• Obesity is one of the main risk factors associated with lethal COVID-19, and levels of pro-inflammatory cytokines increase under this pathology (Michalakis and Ilias, 2020).
• Viral shedding and the production of pro-inflammatory factors is increased during COVID-19 because the adipose tissue has a considerable level of ACE2 expression (Belančić et al., 2020).
• Obesity contributes to thrombotic processes, a probable cause of multiorgan failure, which has been evidenced by the presence of elevated levels of megakaryocytes in COVID-19 autopsies (Campbell et al., 2021).
NLRP3 inflammasome
• SARS-CoV-2 activates inflammasomes, large multiprotein assemblies that are broadly responsive to pathogen-associated cellular insults, leading to secretion of proinflammatory cytokines and an inflammatory form of cell death called pyroptosis (Vora et al., 2021).
• SARS-CoV-2 open reading frame (ORF)-8b interacts with the LRR domain of NLRP3 inflammasome activating IL-1β secretion in THP-1 macrophages (Shi et al., 2019).
• SARS-Cov-2 infection leads to NLRP3 inflammasome activation, caspase-1 cleavage, and the release of IL-1β. This stimulates pyroptosis in peripheral blood mononuclear cells from severe COVID-19 (Rodrigues et al., 2020).

It is important to mention that mRNAs and proteins do not necessarily follow the same expression patterns due to post-transcriptional modifications. However, according to Buccitelli and Selbach, both types of data show a reasonable correlation to reveal exciting biology (Buccitelli and Selbach, 2020). In light of this measured and controlled correlation, we performed transcriptomics and proteomics data integration to reveal therapeutic targets and potential drugs to treat severe COVID-19. The novel coronavirus employs a suite of virulent proteins that interacts with human proteins to extensively rewire the flow of information causing COVID-19 (Kumar et al., 2020; López-Cortés et al., 2021). The human proteins physically associated with SARS-CoV-2 are the first line of host proteins (Gordon et al., 2020), which also interact with proteins involved in a wide spectrum of signaling pathways and biological processes within lung cells. In our study, the network of 111 inflammatory proteins with high-confidence interactions with human proteins physically associated to SARS-CoV-2 proteins showed significantly higher degree centralities in comparison to the inflammatory protein network (Mann-Whitney U test, *p* < 0.05). The top ten proteins with the highest degree centralities were C3, FN1, NFKB1, RPS19, CTSC, HSPD1, APP, ITGAM, SNAP23, and MAPK14.

Subsequently, we analyzed these 111 inflammatory response proteins to identify those with the shortest pathways to physiological phenotypes related to COVID-19. Inflammation is observed in patients with SARS-CoV-2 infection (Saini et al., 2020). The chronic inflammatory process causes cell death (Lee et al., 2020; Li et al., 2020), angiogenesis (Ackermann et al., 2020a), and during the peak of inflammation, immune cells preferentially use glycolysis as a source of energy (Ardestani and Azizi, 2021). These facts provide a biological rationale to analyze and prioritize the inflammatory response proteins with the shortest distance scores to these biological phenotypes. Interestingly, we identified 34 essential inflammatory response proteins highly associated with cell death, glycolysis, and angiogenesis showing not significant difference of distance scores among them (Bonferroni correction test, *p* > 0.1). These proteins were: PTGS2, PRKCZ, NFKBIA, MAPK14, TNFRSF1B, TLR4, ATM, MECOM, PIK3CG, EGFR, JAK2, LYN, CYLD, PRKCQ, STAT3, TGFB1, RBPJ, TNFAIP3, NOTCH1, IGF1, CD28, CCL5, PTAFR, FPR1, EDNRA, EDNRB, CYSLTR1, CNR2, HGF, EPHA2, FN1, CSF1, PTGFR, and APP.

According to Hanahan, inflammation, cell death, glycolysis, and angiogenesis are considered hallmarks of cancer (Hanahan, 2022). However, these biological phenotypes are also observed in COVID-19 patients. Post-mortem lung sections has revealed that pulmonary inflammatory responses may induce infiltration of inflammatory cells that trigger strong immune pathogenesis (Li et al., 2020). Mechanisms of inflammatory response and cell death are strongly linked during SARS-CoV-2 infection (Amaral and Bortoluci, 2020). The infection of lung cells activates caspase-8 to trigger cell death pathways, where apoptosis, pyroptosis, and necroptosis are involved. Interestingly, Vijayakumar et al. (2022) found that post-COVID-19 patients showed abnormal airway proteomes, with elevated concentration of proteins associated with apoptosis. Regarding glycolysis, scientific evidence showed that high glucose concentration and glycolysis are essential for SARS-CoV-2 replication, inflammatory response, and upregulation of ACE2 (Codo et al., 2020). Lastly, vasoconstriction and subsequent hypoxia, stimulate the formation of new blood vessels by promoting branching of pre-existing blood vessels and *de novo* angiogenesis that contributes to the already established systemic hypoxia (Huertas et al., 2020). This process together with the systemic hypoxia observed in severe COVID-19 patients cause a structural and functional reorganization of the pulmonary tissue. Interestingly, Ackermann et al. (2020b) found that in lungs from COVID-19 patients, the amount of new vessel growth was 2.7 times as high as that in lungs from patients with influenza.

Effective therapies are urgently required to treat symptoms and to reduce the mortality caused by SARS-CoV-2 infection. The drug repurposing strategy is the best option to rapidly identify a therapeutic that is effective at improving the clinical outcomes of severe COVID-19 disease (Tejera et al., 2020; López-Cortés et al., 2021). Hence, once we proposed the 34 essential inflammatory response proteins strongly linked to physiological phenotypes of COVID-19, we used the COVID-19 Target Prioritization Tool from the Open Target Platform (Carvalho-Silva et al., 2019) to identify 3 small molecules (baricitinib, eritoran, and montelukast) involved in phases III/IV clinical trials that acts on 3 therapeutic targets (JAK2, TLR4, and CYSLTR1).

Walz et al. (2021) concluded that Janus kinase-inhibitor treatment is significantly associated with positive clinical outcomes in terms of mortality, intensive care unit admission, and discharge. Baricitinib is a tyrosine-protein kinase JAK2 inhibitor mainly used for rheumatoid arthritis, and among its pharmacological properties it has an antiviral effect on the entry of a virus (Cantini et al., 2020). The WHO’s Guideline Development Group found moderate certainty evidence that baricitinib improved survival and reduced the need for ventilation, with no observed increase in adverse effects (Kmietowicz, 2022). Additionally, the WHO strongly recommends baricitinib for patients with severe COVID-19 in combination with corticosteroids (Rochwerg et al., 2020). At the moment, baricitinib is being analyzed in 7 phase III clinical trials with positive clinical outcomes, and has been approved by the WHO, the Food and Drug Administration (FDA) of the United States, and the National Institutes of Health (NIH) for emergent use in severe COVID-19 (COVID-19 Treatment Guidelines Panel, 2020; Kmietowicz, 2022). On the other hand, eritoran is a Toll-like receptor 4/MD-2 antagonist that downregulates the intracellular generation of pro-inflammatory cytokines IL-6 and TNF-alpha in human monocytes. Shirey et al. (2021) examined how antagonizing TLR4 signaling has been effective experimentally in ameliorating acute lung injury and lethal infection in challenge models triggered by acute lung injury-inducing viruses. At the moment, eritoran is being analyzed in 1 phase IV clinical trial with positive clinical outcomes. Lastly, montelukast is a cysteinyl leukotriene receptor 1 antagonist used as part of an asthma therapy regimen whose mechanism blocks the action of leukotriene D4 resulting in decreased inflammation of lung smooth muscle (Wishart et al., 2018). This leukotriene receptor antagonist is being analyzed in 2 phase III clinical trials with positive clinical outcomes.

Considering the enormous pressure that health systems are facing due to the COVID-19 pandemic, it is imperative to recognize the urgent need to diminish the gaps between research and the implementation of public health measures (Park et al., 2021). This is of particular interest to our research given that we acknowledge that clinical trials are essential in evidence-based medicine, and consequently, in the decision making process of public health policies and strategies. Clinical trial networks are essential to coordinate actions between clinical researchers and health practitioners, also promoting knowledge sharing, leadership, and cost-time reductions. In addition, it is critical to decentralize, improve, and increase clinical trials in low and middle-income countries.

The role of health research is fundamental in the response to COVID-19. Analyzing potential drug targets for COVID-19, especially the ones that can serve for severe cases, need an urgent and efficient development of well designed and managed clinical trials. Those which can provide potential interventions that help people to live longer, diminish long-term effects, manage pain and/or possible disabilities. In addition, the possible positive effects on the reduction of hospitalization cost, both at the individual level and in terms of possible savings for the national health system. As another study also mentioned, the potential and benefits of repositioning already approved drugs in COVID-19 clinical trials represent a “potential, prompt, cost-effective, and safe solutions for the public and global health problems, with a human-centered approach” (López-Cortés et al., 2021).

In conclusion, respiratory failure is the leading cause of death in severe COVID-19 and understanding the host inflammatory response at the lungs is imperative to identify actionable targets where specific drugs can work effectively. In our study we identified 34 essential inflammatory proteins with both high-confidence protein interactions and shortest pathways to physiological phenotypes related to COVID-19. Subsequently, we proposed 3 small molecules: baricitinib is a tyrosine-protein kinase JAK2 inhibitor that acts on the JAK2 protein; eritoran is a Toll-like receptor 4/MD-2 antagonist that acts on the TLR4 protein; and montelukast is a cysteinyl leukotriene receptor 1 antagonist that acts on the CYSLTR1 protein. These drugs can be considered for treating severe COVID-19 symptoms after being thoroughly evaluated in COVID-19 clinical trials.

## Data Availability

The datasets presented in this study can be found in online repositories. The names of the repository/repositories and accession number(s) can be found in the article/Supplementary Material.
